# Chiral Nonlinear Enhancement with Opposite Circular Dichroism Empowered by Dual Bound States in the Continuum

**DOI:** 10.3390/ma19112287

**Published:** 2026-05-28

**Authors:** Xinran Liu, Liang Wang, Haoran Meng

**Affiliations:** 1 Changchun Institute of Optics, Fine Mechanics and Physics, Chinese Academy of Sciences, Changchun 130033, China; liuxinran0912@163.com; 2 University of Chinese Academy of Sciences, Beijing 100049, China; 3 School of Science, Lanzhou University of Technology, Lanzhou 730050, China

**Keywords:** metasurface, bound state in the continuum, chirality, nonlinear effect

## Abstract

We present a strategy for achieving precisely controllable circular dichroism (CD) in all-dielectric silicon metasurfaces by exploiting bound states in the continuum (BICs). By employing two topologically protected BIC modes and converting them into circularly polarized eigenstates through oblique illumination, we realize a reversal of maximum chirality without any modification to the metasurface geometry. The resulting CD exhibits opposite signs in two distinct spectral regions and can be flexibly adjusted through engineered structural perturbations. The associated quasi-BIC resonances deliver near-unity CD values (±1), ensuring highly efficient spin-selective transmission. Moreover, this platform enables substantial enhancement of multi-band chiral nonlinear optical responses, where the nonlinear emission becomes strongly dependent on the incident spin state across different frequency bands. Based on effective nonlinear efficiency, a sensitive refractive index sensor can be designed. This work offers a versatile route for tailoring extrinsic chirality in achiral metasurfaces and provides a promising foundation for multifunctional chiral photonic devices in applications such as biosensing, chemical detection, and advanced nonlinear optics.

## 1. Introduction

Chirality is a key concept spanning diverse scientific fields and refers to structures that cannot be brought into congruence with their mirror images through any combination of rotations or translations [1]. Driven by its critical role in controlling light–matter interactions, chiral optics has become increasingly prominent, with applications covering negative-index behavior [2,3], biochemical sensing and molecular diagnostics [4,5,6], optical communication technologies [7], advanced imaging methodologies [8,9,10], and next-generation photodetectors [11]. Among current research efforts, dynamically modulating chiral optical responses—particularly in multilayer hybrid metamaterial configurations—has emerged as an active area of interest [12]. Nonetheless, natural materials generally exhibit extremely weak chiroptical signatures, which substantially limits their direct use in practical photonic systems. To enhance these responses, a wide variety of artificially engineered structures featuring intrinsic chirality have been developed, including chiral nanoscale geometries [13], chiral photonic crystals [14], fish-scale-type metamaterials [15,16], reciprocal chiral systems [17], and mechanically reconfigurable designs such as folded and origami-inspired metasurfaces [18,19,20]. Interestingly, strong chiral optical effects can also arise in geometrically achiral structures through extrinsic chirality [21]. In such cases, mirror-symmetric structures acquire an effective chiral response when illuminated under conditions that break system-level mirror equivalence, enabling robust chiroptical behavior without requiring inherently chiral geometries. Due to their structural simplicity, fabrication friendliness, and broad application potential [22,23,24], extrinsically chiral platforms have become an attractive and practical pathway for realizing strong chiral optical functionalities.

In recent years, a distinctive class of localized resonant states—referred to as bound states in the continuum (BICs)—has drawn widespread interest across multiple research disciplines [25,26,27,28,29,30]. Originally proposed by von Neumann and Wigner in 1929 in the context of quantum mechanics [31], BICs have since been experimentally observed in numerous physical platforms [32,33,34]. Within photonics, BICs are generally categorized into two main families: Friedrich–Wintgen BICs [35,36], which originate from destructive interference among different radiative channels [37], and symmetry-protected BICs [38], which occur when the symmetry of a confined mode prevents coupling to the surrounding continuum. Although ideal BICs exhibit theoretically infinite quality (Q) factors and vanishing linewidths—rendering them non-radiative and thus inaccessible in practical measurements—small, deliberate perturbations can relax the interference or symmetry conditions, converting these states into quasi-BICs. The resulting quasi-BICs preserve exceptionally high Q factors and typically manifest as sharp Fano resonances. These features have made quasi-BICs indispensable for a range of advanced photonic functionalities, including low-threshold lasing [39], tunable acousto-optic interactions [40], enhanced nonlinear frequency generation [41], wireless energy delivery [42], and directional light emission [43]. Recently, quasi-BICs have also emerged as an effective mechanism for tailoring chiroptical responses in metamaterials [44,45,46,47,48,49,50,51,52,53,54,55,56,57,58,59,60,61,62,63,64,65,66]. For example, Gorkunov et al. demonstrated that lifting out-of-plane mirror symmetry in intrinsically chiral metasurfaces gives rise to high-Q quasi-BIC resonances, thereby enabling strong microwave chiral responses [60]. Likewise, Overvig et al. showed that vertically stacked and rotationally offset chiral metasurfaces can sustain chiral quasi-BIC modes with tunable optical chirality [61]. However, current studies on chiral quasi-BIC phenomena predominantly rely on bilayer structures [48,59]; whether this effect can be realized within planar resonant units—thereby avoiding the complexities of 3D structural design—remains a subject for further investigation.

In this work, we demonstrate a planar metasurface platform capable of generating pronounced extrinsic chirality with opposite circular dichroism (CD) signs across multiple spectral bands. The strong extrinsic chiral response stems from system-level mirror-symmetry breaking, which is induced by the asymmetric coupling between incident electromagnetic waves and an otherwise achiral metasurface. This asymmetry is substantially strengthened through the excitation of two distinct quasi-bound states in the continuum (quasi-BICs). By exploiting the topological features associated with BICs, the metasurface lifts the polarization singularity and supports a pair of elliptical polarization eigenmodes. Their selective coupling with circularly polarized excitation gives rise to near-ideal extrinsic CD, enabling efficient spin-dependent optical interactions. A key outcome of this mechanism is the appearance of CD extrema with opposite signs at two separate resonance wavelengths, which facilitates functionalities such as multiband polarization filtering, spin-controlled transmission, and potential cross-band mode interactions. As a proof of concept, we realize third-harmonic generation (THG) that is strongly dependent on the helicity of the incident light in two distinct spectral bands. Based on effective nonlinear efficiency, a sensitive refractive index sensor can be designed. This study establishes a general and robust route to achieving strong, tunable extrinsic chirality and enhanced chiral nonlinearities in achiral photonic structures, offering promising opportunities for multiband spin-selective transmission, biochemical sensing, and integrated chiral photonic technologies.

## 2. Materials and Methods

**Figure 1:**
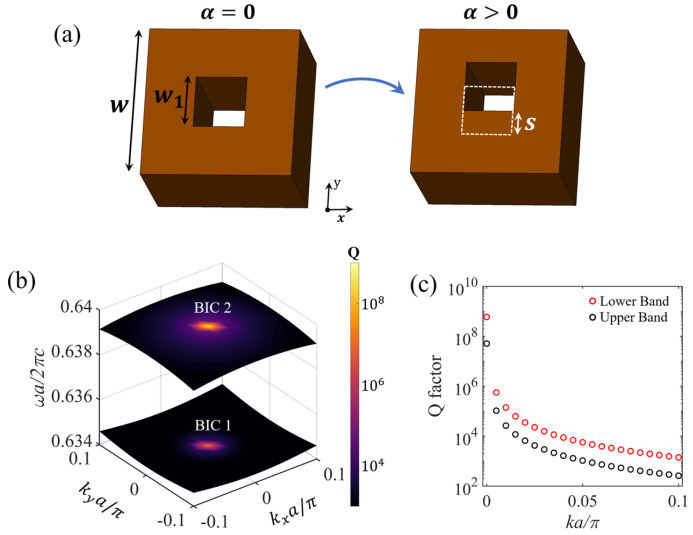
(**a**) Schematic of the unit cell of chiral metasurface. (**b**) The calculated band structure and (**c**) Q factor of two bands with α=0.

As depicted in Figure 1a, the metasurface is formed by a square lattice with a period of a=1010 nm. Each unit cell incorporates a silicon pillar characterized by a width w=620 nm, an air-hole width $w_{1}=180\;nm$, and a thickness t=435 nm. A lateral displacement of the air hole along the y-direction by a distance s (α=s/a) introduces a controlled perturbation that breaks the in-plane symmetry of the otherwise symmetric unit cell. In the absence of such perturbation, the structure preserves a *C*_4*v*_ point-group symmetry, supporting symmetry-protected bound states in the continuum (BICs) at the Γ point—the center of the Brillouin zone. Beyond lateral hole displacement, the structure may also be tilted to break out-of-plane mirror symmetry, as illustrated in Figure 1a. Figure 1b presents the photonic band diagram for the unperturbed case (α=0). The color mapping represents the Q factors of the corresponding eigenmodes, which diverge at the BIC conditions. With the full symmetry preserved, the symmetry-protected BICs remain fixed at the Γ point. Two such BIC modes, labeled BIC1 and BIC2, appear at normalized frequencies ωa/2πc=0.634 and 0.639, respectively. When the wavevector deviates from the Γ point, the Q factors of these modes drop sharply by several orders of magnitude, as shown in Figure 1c, emphasizing the strong momentum-space sensitivity of BICs and their susceptibility to even minor perturbations.

## 3. Discussion

**Figure 2:**
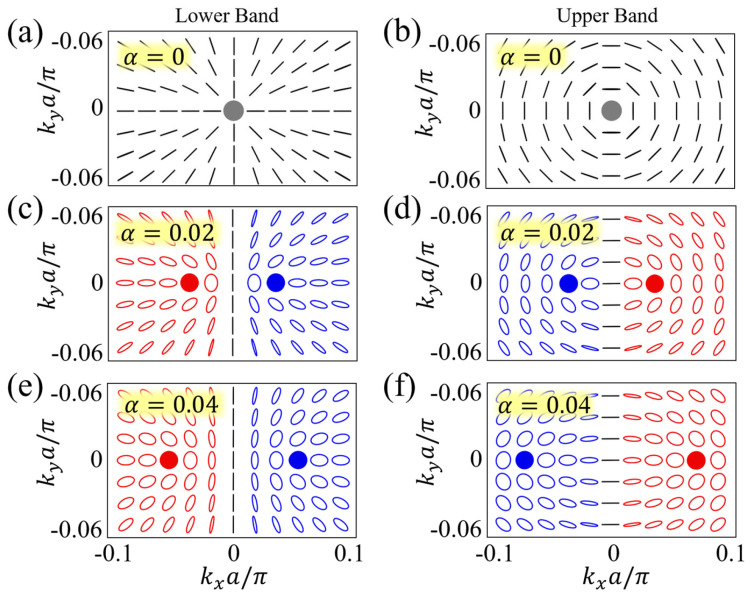
The far-field polarized state distribution evolution in momentum space. Left column: lower band with (**a**) α=0, (**c**) α=0.02, and (e) α=0.04. Right column: upper band with (**b**) α=0, (**d**) α=0.02, and (**f**) α=0.04.

The far-field polarization profiles near the BICs are computed and shown in Figure 2a. At the BICs, a polarization singularity arises, where the polarization direction becomes undefined. This indeterminacy is responsible for the decoupling of the BICs from the surrounding continuum states. The optical vortex at the BICs can be characterized by the topological charge q, defined as [67]

(1) $$q=\frac{1}{2\pi }\oint_{C}dk\cdot \nabla_{k}\phi \left(k\right).$$

Here, *C* represents a closed, simple path in *k*-space that encircles the vortex center in a counterclockwise direction. The polarization angle is given by $\phi \left(k\right)=arg[C_{x}\left(k\right)+iC_{y}(k)]$, where $C_{x}\left(k\right)$ and $C_{y}\left(k\right)$ denote the electric field components along the *x*- and *y*-axes, respectively. In the region surrounding the BICs, the radiative polarization vectors exhibit an almost purely linear form, arranged in a characteristic vortex distribution, as illustrated in Figure 2a,b. Precisely at the BIC positions, a polarization singularity arises, where the far-field polarization state becomes ill-defined owing to the complete elimination of radiation leakage. This singular point constitutes a topological defect in the polarization field, in agreement with the theoretical understanding that bound states in the continuum are intrinsically linked to vortex-type polarization textures. To elucidate the mechanism underlying the emergence of chirality in this metasurface, we examine how the polarization singularities evolve within momentum space. Figure 2c,d illustrate the far-field polarization distributions of the first and second bands, respectively, for a perturbation of α=0.02. In these mappings, red and blue correspond to right-handed (RCP) and left-handed (LCP) circular polarization components. Introducing a finite displacement *s* breaks the original *C*_4*v*_ rotational symmetry of the system, causing the formerly symmetry-protected BICs to split. This symmetry reduction generates a pair of polarization singularities exhibiting opposite circular polarization handedness. Importantly, the separated RCP and LCP states propagate along different directions in the two photonic bands, a feature that is essential for achieving extrinsic chirality reversal.

**Figure 3:**
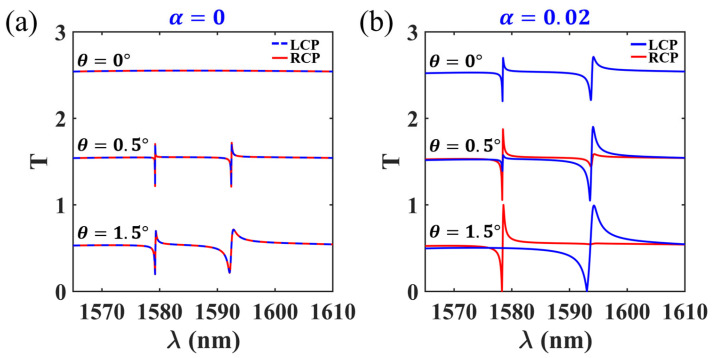
(**a**) The transmission spectrum of the two circularly polarized states with non-zero θ. (**b**) The transmission spectrum of the two circularly polarized states with non-zero α and θ.

Figure 3a displays the transmission spectra for RCP and LCP excitations under different perturbation conditions, allowing us to trace the evolution from ideal BICs to quasi-BIC resonances. The spectra are computed for varying values of the asymmetry parameter α and incident angle *θ*, where θ  represents the angle between the incident wavevector and the surface normal along the x-direction. When α=0  and θ=0°, the structure maintains its full symmetry, and the BICs remain pinned at the Γ point, completely decoupled from radiative channels. In this regime, no resonant features appear in the transmission response, as the incident light cannot excite these non-radiative modes—an indication of an ideal BIC with vanishing far-field signature. Once θ becomes nonzero, symmetry is broken and sharp resonance peaks emerge, signifying the activation of quasi-BICs. The mechanism responsible for this activation differs depending on the type of symmetry breaking.

Importantly, neither of the symmetry-breaking mechanisms—altering α or θ alone—can induce chirality in the system. A chiral optical response is only observed when both parameters are simultaneously nonzero, as demonstrated in Figure 3b. Under this condition, the system exhibits pronounced chiral behavior. By fixing the structural displacement at α=0.02 and gradually increasing θ, the chirality of the quasi-BICs is progressively enhanced. Notably, at θ=1.5°, the system reaches its maximal opposite extrinsic chirality at two distinct wavelengths. It is worth mentioning that when α is reversed, the chirality will be reversed, as shown in Figure 3b.

**Figure 4:**
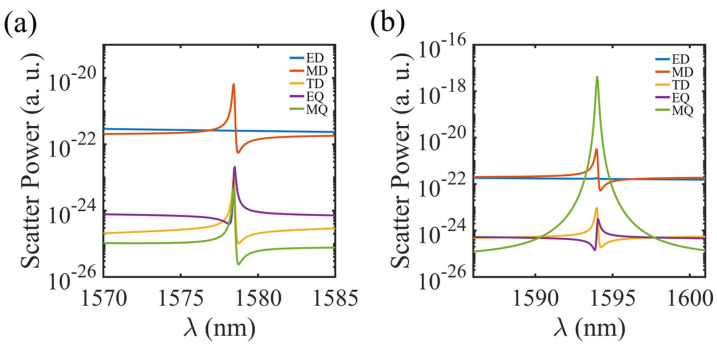
Contributions of the different multipolar excitations of the two quasi-BIC on (**a**) upper band and (**b**) Lower band.

Based on the multipole scattering theory, the scattering powers of different multipole moments induced in the metasurface with α=0 and θ=1.5° is shown in Figure 4. The incident polarization state is left-handed polarization. According to the general multipole scattering theory, the total intensity of scattering power can be defined as

(2) $$I=\frac{2\omega^{4}}{3c^{3}}{\left|P\right|}^{2}+\frac{2\omega^{4}}{3c^{3}}{\left|M\right|}^{2}+\frac{4\omega^{5}}{3c^{4}}{\left|P\cdot T\right|}^{2}+\frac{2\omega^{6}}{3c^{5}}{\left|T\right|}^{2}+\frac{\omega^{6}}{5c^{5}}\sum {\left|Q_{\alpha \beta }\right|}^{2}+\frac{\omega^{6}}{40c^{5}}\sum {\left|M_{\alpha \beta }\right|}^{2},$$

where P, M, T, $Q_{\alpha \beta }$, and $M_{\alpha \beta }$ are the electric dipole (ED), magnetic dipole (MD), toroidal dipole (TD), electric quadrupole (EQ), and magnetic quadrupole (MQ), respectively. It can be seen that one is dominated by the MD mode, while the other is dominated by the MQ mode.

**Figure 5:**
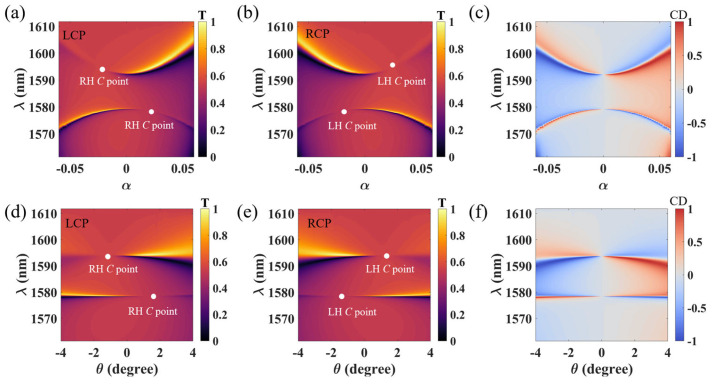
Transmission spectra with θ=1.5° versus α for (**a**) LCP and (**b**) RCP waves. (**c**) CD spectrum of the structure versus incident angle. Transmission spectra with α=0.02 versus incident angle for (**d**) LCP and (**e**) RCP waves. (**f**) The CD spectrum of the structure versus incident angle.

Figure 5 presents a qualitative analysis of the CD exhibited by the structure. Figure 5a,b show the transmission spectra for LCP and RCP incident light, respectively, under varying structural perturbation α, with the angles of incidence θ  at 1.5°. In Figure 5a, the resonance linewidth vanishes when α=0.02 in the lower band and when α=−0.02 in the upper band, corresponding to a right-handed circular (RH *C*) polarization state. Conversely, in Figure 5b, the resonance linewidth vanishes when α=−0.02 in the lower band and when α=0.02 in the upper band, corresponding to a left-handed circular (LH *C*) polarization state.

The CD value is described by the different response of the proposed structure to the LCP and RCP wave. The CD can be described as

(3) $$CD=\frac{{\left|t_{R}\right|}^{2}-{\left|t_{L}\right|}^{2}}{{\left|t_{R}\right|}^{2}+{\left|t_{L}\right|}^{2}},$$

in which $t_{R}$ denotes the transmission coefficient of the RCP light, $t_{L}$ is the transmission coefficient of the LCP light, respectively. To provide a more detailed quantification of chirality, Figure 5c displays the calculated CD values as a function of the structural perturbation α. At the two quasi-BIC wavelengths, the CD approaches an ideal value of 1, indicating near-perfect chirality. This reversal of the CD sign reveals the strongly tunable chirality of the system. We compared the performance of chiral dual BICs with previous work as shown in Table S1 of Supplementary Materials.

We further calculated the transmission spectrum with different incident angle θ at fixed α=0.02. In Figure 5d, the resonance linewidth vanishes when θ=1.5° in the lower band and when θ=−1.5° in the upper band, corresponding to a right-handed circular (RH *C*) polarization state. Conversely, in Figure 5e, the resonance linewidth vanishes when θ=−1.5° in the lower band and when θ=1.5° in the upper band, corresponding to a left-handed circular (LH C) polarization state. This symmetry can be attributed to the distinct distributions of circularly polarized states in momentum space, which result in polarization-dependent behavior of the reflection spectra as the incident angle varies.

Building on this foundation, we employ the dual quasi-BIC modes discussed above to realize chiral nonlinear enhancement in metasurfaces. Owing to its large third-order nonlinear susceptibility, silicon is a highly suitable material platform for nonlinear photonics. The degree of nonlinear enhancement is closely tied to how strongly the electromagnetic fields are confined within the structure. Consequently, quasi-BIC modes—capable of significantly reducing radiative losses—offer an effective route for boosting optical nonlinear responses. We selected α=0.02. At this time, the maximum extrinsic chirality exists in the dual bands at the oblique incident angle θ=1.5°, as shown in Figure 5. The THG efficiency is defined as

(4) $$\eta =\frac{\iint_{A}^{0}{\vec{S}}_{TH}\cdot \hat{n}\;da}{I_{0}},$$

where ${\vec{S}}_{TH}$ represents the Poynting vector of the field, $\hat{n}$ is the unit vector normal to a surface A enclosing the cylinder, and $I_{0}$ is the pump intensity. The nonlinear optical simulations using COMSOL6.0 Multiphysics in the frequency domain. The pump intensity as $0.5\;MW/{cm}^{2}$, and incident angle θ=1.5°.

**Figure 6:**
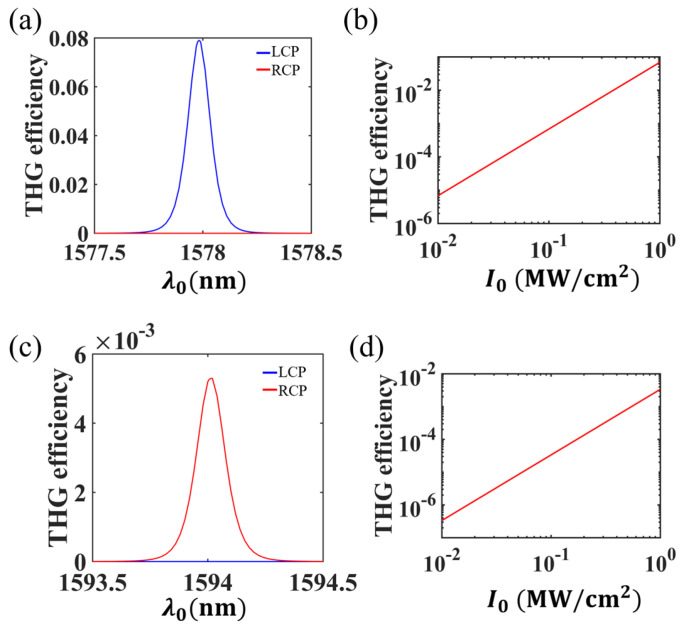
(**a**) The THG efficiency of low-frequency BIC under different circularly polarized excitations and (**b**) its dependence on pump intensity. (**c**) The THG efficiency of high-frequency BIC under different circularly polarized excitations and (**d**) its dependence on pump intensity.

Next, we evaluated how the low-frequency quasi-BIC enhances third-harmonic generation (THG) under circularly polarized illumination, as illustrated in Figure 6a. A pronounced THG response is obtained for left-circularly polarized (LCP) excitation, exhibiting an efficiency nearly two orders of magnitude higher than that produced by right-circularly polarized (RCP) incidence. This substantial contrast arises from the presence of an extrinsic chiral quasi-BIC with near-ideal chiral selectivity. Because nonlinear optical processes are strongly intensity-dependent, we further analyzed the influence of pump intensity ($I_{0}$) on the THG performance of the metasurface. The peak THG efficiency under LCP excitation is extracted and plotted in Figure 6b. As the pump intensity increases, the THG efficiency shows an exponential growth trend, indicating that proper tuning of the incident intensity enables optimal chiral THG outputs. We also examined the nonlinear enhancement associated with the other quasi-BIC mode. Owing to the reversed chirality of this resonance, a significant THG enhancement occurs under RCP illumination, far exceeding that of the LCP case, as shown in Figure 6c. The dependence of the THG efficiency on pump intensity for RCP excitation is similarly presented in Figure 6d.

**Figure 7:**
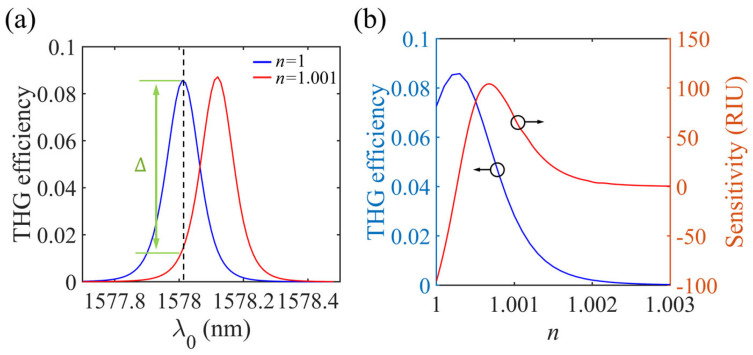
(**a**) The THG efficiency at different environmental refractive indices n. (**b**) The THG efficiency (the blue line) of the proposed sensor as a function of the environmental refractive index *n*. The red line represents the index-dependent sensitivity of the sensor.

Based on effective nonlinear efficiency, a sensitive refractive index sensor can be designed. The sensor based on α=0 and θ=1.5° under LCP wave incidence. We investigate the influence of a slight change in the refractive index n around the metasurface on its nonlinear efficiency, as shown in Figure 7a. It can be seen that a tiny change in the surrounding refractive index leads to an offset in the THG efficiency Δ. Therefore, the sensitivity of the proposed sensor can be defined as the derivative of the THG efficiency at fixed wavelength $\lambda_{0}=1578.02\;nm$ concerning the refractive index, that is,

(5) $$S=-\frac{d\eta }{dn}.$$

The sensitivity of the proposed sensor is visually denoted by the red line in Figure 7b. The proposed sensor has a maximum sensitivity of 102 RIU, indicating that it can be used to detect tiny variations in the environmental refractive index, such as climate monitoring, biochemical detection, and foreign object detection.

## 4. Conclusions

In summary, we demonstrate strong extrinsic chirality manifested by opposite circular dichroism (CD) responses in distinct frequency regions. By introducing structural asymmetry into a metasurface supporting dual BIC modes, we generate chiral quasi-BICs whose handedness reverses between the two resonant bands. This chirality inversion originates from the fact that circularly polarized states experience different splitting behaviors across the bands. Leveraging the reversed chirality at separate resonances, we achieve spin-dependent third-harmonic generation (THG) in both bands. The high nonlinear conversion efficiency also allows the design of a highly sensitive refractive-index sensor. Overall, this work extends the capabilities of BIC-based metasurfaces for enhancing extrinsic chirality and provides a foundation for advanced applications such as multiband spin-selective photonic devices, optical encryption, and on-chip manipulation of chiral light.

## Data Availability

The original contributions presented in this study are included in the article/Supplementary Materials. Further inquiries can be directed to the corresponding author.

## Supplementary Materials

The following supporting information can be downloaded at: https://www.mdpi.com/article/10.3390/ma19112287/s1, Table S1: Performance Comparison of Chiral Dual-BICs.
