# Supplementary material for: Chiral Nonlinear Enhancement with Opposite Circular Dichroism Empowered by Dual Bound States in the Continuum
Source: Materials (Basel). 2026 May 28;19(11):2287. doi: 10.3390/ma19112287 (PMC13257638; doi:10.3390/ma19112287)
Supplement: Supplementary file 1 [file materials-19-02287-s001.zip › materials-4301207-supplementary.pdf]

Chiral nonlinear enhancement with opposite circular dichroism empowered by Dual bound states in the continuum

Xinran Liu[1,2], Liang Wang[3] and Haoran Meng[1,*]

[1] Changchun Institute of Optics, Fine Mechanics and Physics, Chinese Academy of Sciences, Changchun, China
[2] University of Chinese Academy of Sciences, Beijing, China
[3] School of Science, Lanzhou University of Technology, Lanzhou, China
* Correspondence: menghaoran@ciomp.ac.cn

S-1. Performance Comparison of Chiral Dual-BICs

Here, we compared the present work with our previous studies on bi-chirality and summarized the findings in a table, as follow:

Ref.	Structure	CD	Chirality of Dual BICs	Q factor
[48]	double-layer	0.7	opposite	~7000
[59]	double-layer	1	same	~500
this work	single-layer	1	opposite	~1500

Table S1. Performance Comparison of Chiral Dual-BICs.
